# The effect of maternal vitamin D deficiency during pregnancy on glycolipid metabolism of offspring rats and the improvement of vitamin D intervention after weaning

**DOI:** 10.3389/fnut.2023.1214040

**Published:** 2023-07-31

**Authors:** Zhaojun Chen, Yunxia Zhu, Ting Wu, Xia Qian, Ye Hu, Wensheng Hu

**Affiliations:** Department of Child Health Care, Hangzhou Women’s Hospital (Hangzhou Maternity and Child Care Hospital), Hangzhou, China

**Keywords:** vitamin D deficiency, body weight, lipid metabolism, glucose metabolism, offspring, pregnancy, rats

## Abstract

**Background:**

Vitamin D deficiency during pregnancy is common, but whether maternal vitamin D status affects glycolipid metabolism of offspring remains unclear.

**Objective:**

To evaluate the effect of maternal vitamin D deficiency during pregnancy on the glycolipid metabolism of offspring at different life-cycles (from birth to adulthood) and to explore the improvement of different dosages of vitamin D supplementation.

**Methods:**

Sprague–Dawley rats were fed vitamin D-deprived (VDD group) or standard vitamin D diets (SC group) during pregnancy, and their diets were changed to standard vitamin D diets during lactation (the offspring were sorted into VDD_offspring_ and SC_offspring_ groups). After weaning, rats in the VDD_offspring_ group were randomly assigned to the VDD_offspring_, VDD_offspring-_S_3300_ and VDD_offspring-_S_10000_ groups with diets containing standard, medium and high dosages of vitamin D for 12 wk. Serum was collected for biochemical analyses at postnatal Day 21, postnatal Day 56 and postnatal Day 84. Oral glucose tolerance test (OGTT) was performed at postnatal Day 70.

**Results:**

Compared to SC_offspring_, rats in the VDD_offspring_ group had significantly lower birth weight with faster weight gain and higher levels of lipid metabolism in early life. After near adulthood, the differences in weight and lipid metabolism between the two groups disappeared. OGTT showed significantly higher blood glucose levels in the VDD_offspring_ group at 30 min, 60 min, and 90 min. The continuation of vitamin D supplementation at medium and high dosages after weaning did not cause any obvious changes in weight or glycolipid metabolism (except for postprandial hyperglycemia). OGTT demonstrated that the glucose levels in the VDD_offspring_-S_3300_ group were lowest at all the time points and that those in the VDD_offspring_-S_10000_ group were the highest at 30 min, 60 min, and 90 min among the three groups.

**Conclusion:**

The adverse effects of vitamin D deficiency during pregnancy on glycolipid metabolism in offspring vary in different stages. Over a long time period, adequate vitamin D supplementation is beneficial to glycolipid metabolism for the offspring of subjects with vitamin D deficiency during pregnancy; however, further improvement is required.

## Introduction

Vitamin D, which is a prohormone, not only plays an important role in calcium and phosphorus homeostasis and bone health (1), but also exerts pleiotropic actions regarding cellular differentiation and proliferation, immune regulation and metabolism (2, 3). Sufficient vitamin D is generally considered a protective factor for maintaining good health and for preventing diseases. Notably, maternal vitamin D deficiency during pregnancy (25-hydroxyvitamin D [25(OH)D] < 30 nmol/L) is very common, even in areas with high ultraviolet exposure, which has been observed as a worldwide public health problem (4, 5). Thus, more attention has been focused on the role of vitamin D during pregnancy.

Recently, the adverse effects of vitamin D deficiency on glycolipid metabolism during pregnancy has been reported by numerous studies, including elevated risks for gestational diabetes mellitus (GDM), higher blood glucose and dyslipidemia in pregnancy (6–10). Furthermore, fetal and neonatal vitamin D status completely relies on the conditions of the mother (11), and there is a question as to whether vitamin D deficiency during pregnancy has an effect on glycolipid metabolism in the offspring. However, related studies have been poorly investigated. A maternal and infant study found that vitamin D-deficient women had higher concentrations of triglycerides and lower concentrations of high-density lipoprotein cholesterol (HDL-C) and Homeostasis model assessment- β (HOMA-β) in infant umbilical arterial blood in comparison with pregnant women with adequate vitamin D. Additionally, the triglyceride level in the umbilical artery was negatively correlated with maternal serum 25(OH)D concentration (12). Another cohort study with 1882 mother–child pairs showed that a 10 nmol/L increase in maternal 25(OH)D was associated with a 0.02 (99% CI: −0.03, −0.004) decrease in Homeostatic model assessment of insulin resistance (HOMA-IR), and a 0.13% (99% CI: −0.3, −0.003) decrease in body fat percentage at the approximate age of 5-6-years-old (13). Nevertheless, a study from India indicated that in addition to higher fasting insulin resistance at the age of 9.5 years, maternal vitamin D deficiency during pregnancy did not affect blood glucose and fasting lipid levels in the offspring at age 5 or 9.5 (14). An observational cohort study followed 149 healthy pregnant women pointed maternal vitamin D levels did not correlate with neonatal serum glucose or insulin levels (15). To date, all of the results of relevant studies are inconsistent and conflicting (5, 16, 17). These differences may be explained by several confounding factors including sample size, geographical location, gestational age at sampling, end time of observation indicator and the rate of vitamin D deficiency (16, 17).

Moreover, most research has mainly emphasized the effects at the early stages of offspring (neonatal or infant stages). According to the Developmental Origins of Health and Disease (DOHaD) theory, the effects of adverse nutritional environment *in utero* on fetal heath are long-term and even irreversible (5, 18). Additionally, some other studies concerning the association between vitamin D deficiency during pregnancy and lipid metabolism of their offspring were performed based on obesity diet models, which face difficulties in elucidating the independent effects of vitamin D on lipid metabolism (19–21). To better control the confounding factors, we constructed a prenatal vitamin D deficient rat model based on a normally structured diet (only vitamin D deficiency or not) to assess the independent effect of maternal vitamin D deficiency during pregnancy on the glycolipid metabolism of their offspring at different life cycles (from birth to adulthood). Moreover, the offspring with vitamin D deficiency during pregnancy were given different dosages of vitamin D supplementation (standard, medium and high dosages) after weaning, in an attempt to explore whether vitamin D supplementation in offspring could promote the adverse effects of vitamin D deficiency during pregnancy.

## Methods

### Animal study

Sprague–Dawley rats (7-weeks-old, Shanghai SLAC Laboratory Animal Co., Ltd) were housed in incandescent light devoid of ultraviolet B radiation on a 12 h light/12 h dark cycle at 22 ± 2°C and 60% relative humidity. They were randomly divided into a standard group and a vitamin D-deficient group according to initial weights. The sufficient group (SC; n = 4) was fed a standard vitamin D diet containing 1,000 IU vitamin D3/kg (AIN-93G with 1,000 IU vitamin D_3_/kg, Trophic Animal Feed High-tech Co., Ltd., China), and the vitamin D-deficient group (VDD; n = 8) was fed a vitamin D-deprived diet containing 25 IU vitamin D_3_/kg (AIN-93G with 25 IU vitamin D_3_/kg, Trophic Animal Feed High-tech Co., Ltd., China). After a 4-week deficiency induction period, serum 25(OH)D concentrations were detected to ensure that the vitamin D-deficient model was successfully established. Afterwards, all rats were mated with standard vitamin D diet-fed male SD rats (three rats and seven rats were matched successfully in the SC group and VDD group, respectively). During gestation, two groups of pregnant rats continued to be fed their previous diets, and serum 25(OH)D concentrations were detected on embryonic Day 18 (E18). During lactation, all of the dams in both groups were fed a standard vitamin D diet. At postnatal Day 21 (PND 21, weaning), 8 offspring rats were killed in both groups (SC_offspring_ and VDD_offspring_ groups) for blood sampling, after which we randomly selected 16 and 48 offspring rats from the SC_offspring_ group and VDD_offspring_ group, respectively, for subsequent experiments. Forty-eight offspring rats in the VDD_offspring_ group were randomly assigned to the VDD_offspring_, VDD_offspring_-S_3300_ and VDD_offspring_-S_10000_ groups according their body weights (VDD_offspring_: 49.46 ± 1.00 g; VDD_offspring_-S_3300_: 49.76 ± 1.74 g and VDD_offspring_-S_10000_: 49.26 ± 1.35 g, *p* > 0.05), which were fed a diet containing 1,000 IU vitamin D_3_/kg (standard dosage), 3,300 IU vitamin D_3_/kg (medium dosage) and 10,000 IU vitamin D_3_/kg (high dosage) (AIN-93G with 1,000/3,300/10,000 IU vitamin D_3_/kg, Trophic Animal Feed High-tech Co., Ltd., China) until the end of the experiment. To examine the biochemical index changes, 8 offspring rats were euthanized in four groups for blood sampling at week 8 (PND 56, near adulthood) and week 12 (PND 84, adulthood). All of the offspring rats were weighed on PND 1, PND 3, PND 7, PND 14, PND 21, PND 28, PND 42, PND 56, PND 70 and PND 84.

All experimental procedures followed the guidelines for the care and use of animals, which were established by Zhejiang Chinese Medical University and approved by the Animal Experimentation Ethics Committee of Zhejiang Chinese Medical University (Approval NO. IACUC-20200518-15).

### Vitamin D measurements

Serum was collected after centrifugation of blood samples at 3,500 × g for 10 min and stored at −80°C until analysis, which was performed by using liquid chromatography–mass spectrometry (LC–MS/MS) as previously described (22).

### Fasting variables

500 microliters of blood were collected from the lower mandibular vein after 12 h of fasting，and the serum was separated by centrifugation at 3000 rpm for 10 min. Then the concentrations of calcium, triacylglycerols (TGs), total cholesterol (TC), high-density lipoprotein (HDL), low-density lipoprotein (LDL) and fasting glucose (FG) were measured by using a fully automatic biochemical analyzer (Hitachi 3,100, Japan). The levels of fasting insulin (FIN) in the serum were determined via enzyme-linked immunosorbent assay kits (CUSABIO, Wuhan, China) according to the manufacturer’s guidelines.

### Oral glucose tolerance test

The oral glucose tolerance test (OGTT) was performed in the 10th week after birth. Two-hour OGTT with gavage of 20% glucose (2.0 g/kg glucose solution) were given in groups, followed by collection of blood samples from tail vein at 0. 30, 60, and 120 min to measure plasma glucose levels by an automatic glucometer (Accu-Chek, Roche Diagnostics, Mannheim, Germany).

### Statistical analysis

All of the statistical analyses were performed by using SPSS 23.0 software. Continuous variables were summarized as the mean with standard deviation (SD). Independent samples t tests were used to compare the differences in variables between two groups. One-Way ANOVA analysis and LSD test were applied to compare the differences among multiple groups (more than two groups). Pearson’s correlation analysis was used to examine correlations between different variables (maternal vitamin D level on E18 and lipid metabolism). Moreover, a simple linear regression was used to predict the effects of maternal vitamin D level (E18) on glycolipid metabolism in the offspring. Two-tailed *p* values<0.05 were considered to be statistically significant.

## Results

### Maternal and offspring vitamin D status and calcium levels

**Figure 1:**
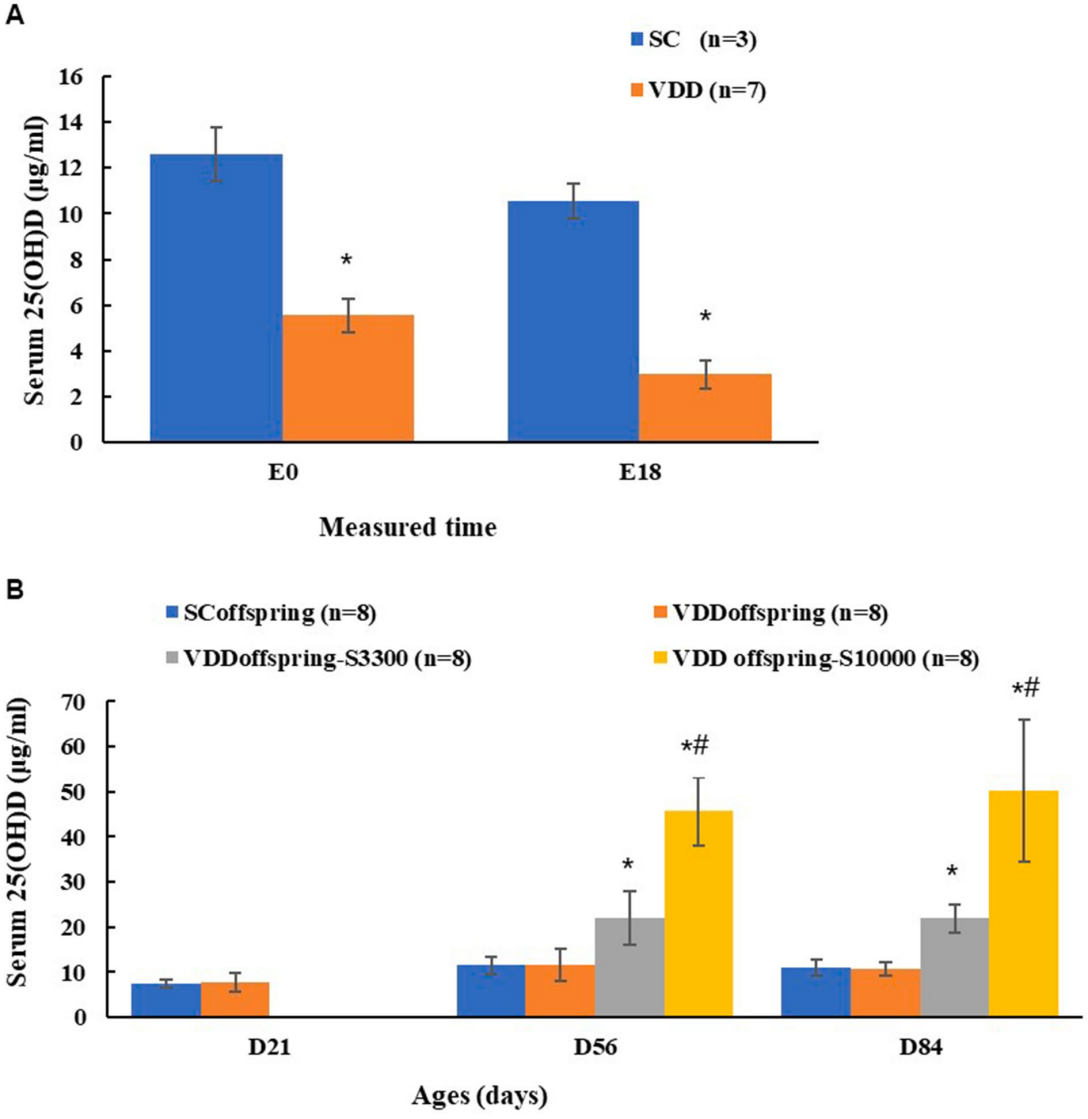
Maternal and their offspring vitamin D status at different time **(A)** Maternal 25(OH)D conentration of two groups on embryonic day 0 (E0) and embryonic day 18 (E18). *Compared to SC group, *p* < 0.05; **(B)** The 25 (OH) D conentration of offspring among four groups on PND21, PND56 and PND84. *Compared to SC_offspring_ group, *p* < 0.05; ^#^Compared to VDD_offspring-S3300_ group, *p* < 0.05.

Maternal 25(OH)D was measured on embryonic Day 0 (E0) and embryonic Day 18 (E18) to determine whether the prenatal vitamin D deficiency model was successfully established in rats. There were significant differences in the concentrations of maternal 25(OH)D between the SC group and VDD group on E0 (12.58 ± 1.18 μg/mL and 5.55 ± 0.75 μg/mL, respectively; *p* < 0.001) and on E18 (10.54 ± 0.77 μg/mL and 2.97 ± 0.59 μg/mL, respectively; p < 0.001), whereas the calcium levels were similar between the SC group and VDD group on E18 (2.55 ± 0.05 mmol/L and 2.47 ± 0.05 mmol/L, respectively; *p* = 0.058), which indicated the success of the model (Figure 1A).

For offspring, the serum 25(OH)D concentrations between the SC_offspring_ group and the VDD_offsspring_ group were not significantly different (*p* = 0.654) on PND21, with the levels being 7.35 ± 0.85 μg/mL and 7.73 ± 2.19 μg/mL, respectively. After weaning with the intervention of different vitamin D dosages, rats in the SC_offsspring_ group and VDD_offsspring_ group maintained similar 25(OH)D levels, and the concentration of 25(OH)D in VDD_offsspring_-S_3300_ was significantly higher than that in the SC_offsspring_ group and VDD_offsspring_ group. Rats in the VDD_offsspring_-SC_10000_ group had the highest 25(OH)D concentrations on PND 56 and PND 84 (Figure 1B). Furthermore, the calcium levels among all groups remained similar at all of the time points (*p* > 0.05). Data were shown in Supplementary Table S1.

### Body weight

**Figure 2:**
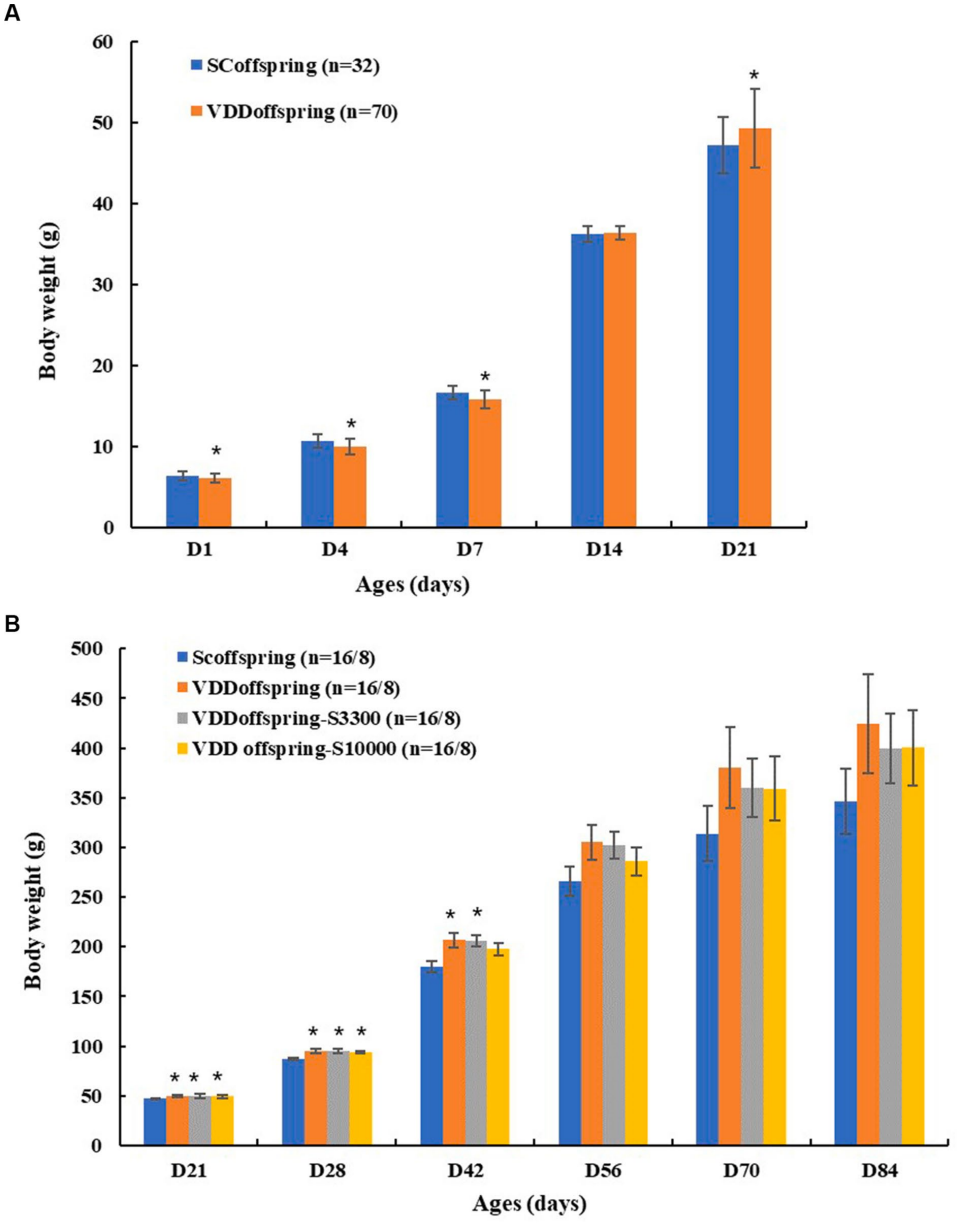
The weight of rats in different groups **(A)** The weight of rats in SC_offspring_ and VDD_offspring_ groups before weaning; **(B)** The weight of rats in SC_offspring,_ VDD_offspring,_ VDD_offspring-_S_3300_ and VDD_offspring-_s_10000_ groups after weaning (from PND21 to PND56, the number of rats in four groups was 16; after PND56, the number of rats in four groups was 8). *Compared to SC_offspring_ group, *p* < 0.05.

As shown in Table 1; Figure 2, there were significant and different effects of maternal vitamin D deficiency during pregnancy on the body weights of their offspring at different life cycles. The birth weights and the weights on PND 4 and PND 7 in the VDD_offspring_ group were significantly lower than those in the SC_offspring_ group (*p* < 0.05); however, the weights of rats in the VDD_offspring_ group seemed to increase at a faster rate. On PND 14, the weights in the two groups were similar; in addition, from PND 21 to PND 42, the body weights in the VDD _offspring_ group were significantly higher than those in the SC_offspring_ group. From the time of PND 56, there was no significant difference in body weights between the two groups; however, the weights in VDD _offspring_ were still higher than those in the SC_offspring_ group.

**Table 1:** Fasting variables among four groups at different time points.

Variable	PND21		PND56				PND84			
	SCoffspring (*n* = 8)	VDDoffspring (*n* = 8)	SCoffspring (*n* = 8)	VDDoffspring (*n* = 8)	VDDoffspring-S3300 (*n* = 8)	VDDoffspring-S10000 (*n* = 8)	SCoffspring (*n* = 8)	VDDoffspring (*n* = 8)	VDDoffspring-S3300 (*n* = 8)	VDDoffspring-S10000 (*n* = 8)
TG (mmol/L)	0.74 ± 0.17	0.92 ± 0.15*	0.74 ± 0.22	0.69 ± 0.47	0.81 ± 0.38	0.46 ± 0.14	0.73 ± 0.12	0.63 ± 0.29	0.59 ± 0.23	0.55 ± 0.13
TC (mmol/L)	2.14 ± 0.21	2.45 ± 0.20**	1.61 ± 0.18	1.76 ± 0.52	1.91 ± 0.34	1.68 ± 0.39	1.68 ± 0.15	1.65 ± 0.31	1.48 ± 0.20	1.52 ± 0.14
HDL-C (mmol/L)	0.62 ± 0.06	0.59 ± 0.04	0.52 ± 0.04	0.54 ± 0.10	0.58 ± 0.05	0.53 ± 0.07	0.58 ± 0.04	0.53 ± 0.04	0.50 ± 0.04	0.52 ± 0.06
LDL-C (mmol/L)	0.45 ± 0.04	0.55 ± 0.07**	0.24 ± 0.05	0.27 ± 0.09	0.29 ± 0.06	0.26 ± 0.09	0.26 ± 0.05	0.26 ± 0.07	0.22 ± 0.05	0.22 ± 0.03
TC/HDL	3.47 ± 0.19	4.15 ± 0.12**	3.08 ± 0.21	3.19 ± 0.34	3.26 ± 0.34	3.12 ± 0.37	2.91 ± 0.27	3.12 ± 0.37	2.95 ± 0.25	2.95 ± 0.22
LDL/HDL	0.72 ± 0.06	0.93 ± 0.09**	0.46 ± 0.07	0.49 ± 0.07	0.50 ± 0.07	0.48 ± 0.10	0.44 ± 0.09	0.48 ± 0.11	0.44 ± 0.07	0.43 ± 0.08
FG (mmol/L)	4.64 ± 0.95	5.11 ± 0.35	4.07 ± 0.38	4.55 ± 0.46	4.67 ± 0.68	4.09 ± 0.85	4.96 ± 0.23	5.29 ± 0.51	5.12 ± 0.91	5.68 ± 0.66^*^
FIN (ng/mL)	5.26 ± 2.22	6.17 ± 2.05	5.43 ± 0.95	4.27 ± 1.53	7.32 ± 2.25^##^	3.73 ± 1.50	3.82 ± 1.83	4.45 ± 1.97	3.37 ± 1.66	2.65 ± 1.39^#^
HOMA-IR	1.03 ± 0.33	1.38 ± 0.39	0.97 ± 0.41	0.88 ± 0.36	1.49 ± 0.42^*##^	0.69 ± 0.37	0.85 ± 0.42	1.06 ± 0.53	0.75 ± 0.33	0.65 ± 0.34

Data are presented as Mean ± SD; *Compared to SC_offspring_ group, *p* < 0.05; **Compared to SC_offspring_ group, *p* < 0.01. ^#^Compared to VDD_offspring_ group, *p* < 0.05; ^##^Compared to VDD_offspring_ group, *p* < 0.01.

TG, triglyceride; TC, total cholesterol; HDL-C, high-density lipoprotein cholesterol; LDL-C, low-density lipoprotein cholesterol; TC/HDL, the ratio of TC and HDL; LDL/HDL, the ratio of LDL and HDL; FG, Fasting glucose; FIN, fasting insulin; HOMA-IR, homeostatic model assessment of insulin resistance.

With medium and high dosages of vitamin D supplementation after weaning, the body weights in the VDD_offspring_-S_3300_ and VDD_offspring_-S_10000_ groups had no significant differences from those in the VDD_offspring_ group at all of the time points (*p* < 0.05). Similarly, compared with SC_offspring_, the weights in the VDD_offspring_-S_3300_ and VDD_offspring_-S_10000_ groups were still significantly higher from PND21 to PND42 (except for the weights in VDD_offspring_-S_10000_ on PND42), after which it maintained a higher weight at later time points.

### Lipid metabolism and related factors

The effect of maternal vitamin D deficiency during pregnancy on the lipid metabolism of the offspring rats was significant in early life. As shown in Table 1, the levels of TG, TC, LDL-C, TC/HDL and LDL/HDL were significantly increased in the VDD_offspring_ group compared with the SC_offspring_ group on PND 21 (*p* < 0.05), and these differences disappeared on PND56 and PND84 (*p* > 0.05).

The continuation of vitamin D supplementation at medium and high dosages after weaning did not cause any obvious change in lipid metabolism among the VDD_offspring_, VDD_offspring_-S_3300_ and VDD_offspring_-S_10000_ groups (*p* > 0.05). We observed similar levels of TG, TC, HDL-C, LDL-C, TC/HDL and LDL/HDL among the three groups on PND56 and PND84.

Pearson correlation analysis showed that maternal vitamin D levels during pregnancy (E18) were negatively correlated with the levels of TC, TG, LDL-C, TC/HDL, and LDL/HDL in their offspring rats on PND21 (Table 2). Additionally, simple linear regression was calculated to determine the effects of maternal vitamin D levels during pregnancy (E18) on the lipid metabolism of offspring rats. We found that 81.4% of the TC/HDL cholesterol ratio and 66.8% of the LDL/HDL cholesterol ratio were determined by maternal vitamin D levels during pregnancy for offspring rats in early life (adjusted *R*^2^ values were 0.814 and 0.668, respectively, *p* < 0.05). Data are shown in Supplementary Table S2.

**Table 2:** Pearson’s correlation coefficients among related factors (maternal VD levels on E18 and VD of offspring on PND21) and lipid metabolism on PND21.

Related factors	TC	TG	HDL	LDL	TG/HDL	LDL/HDL
Maternal VD levels (*n* = 5)	**−0.586** ^*^	**−0.504** ^*^	0.325	**−0.658** ^**^	**−0.902** ^**^	**−0.817** ^**^
VD levels of offspring (*n* = 16)	0.425	0.355	0.318	0.346	0.154	0.133

Bold indicates the relationships are significant statistically. *p < 0.05; **p < 0.01.

### Glucose metabolism and oral glucose tolerance test

Compared with the SC_offspring_ group, no significant differences in glucose metabolism (fasting glucose, fasting insulin and HOMA-IR) were found in the VDD_offspring_ group at all of the time points, although they were numerically higher (Table 1). However, OGTT showed that significantly higher blood glucose levels were detected in the VDD_offspring_ group than in the SC_offspring_ group at 30 min, 60 min, and 90 min, although blood glucose levels at 0 min and 120 min were similar between the two groups (Figure 2). Afterwards, simple linear regression was calculated to determine the effect of maternal vitamin D levels during pregnancy (E18) on the blood glucose levels of offspring rats, which indicated that 35.8, 50.8, and 20.4% of blood glucose levels at 30 min, 60 min, and 90 min were determined by maternal vitamin D levels during pregnancy, respectively (adjusted *R*^2^ values were 0.358, 0.508, and 0.204, respectively, *p* < 0.05). Data are shown in Supplementary Table S2.

**Figure 3:**
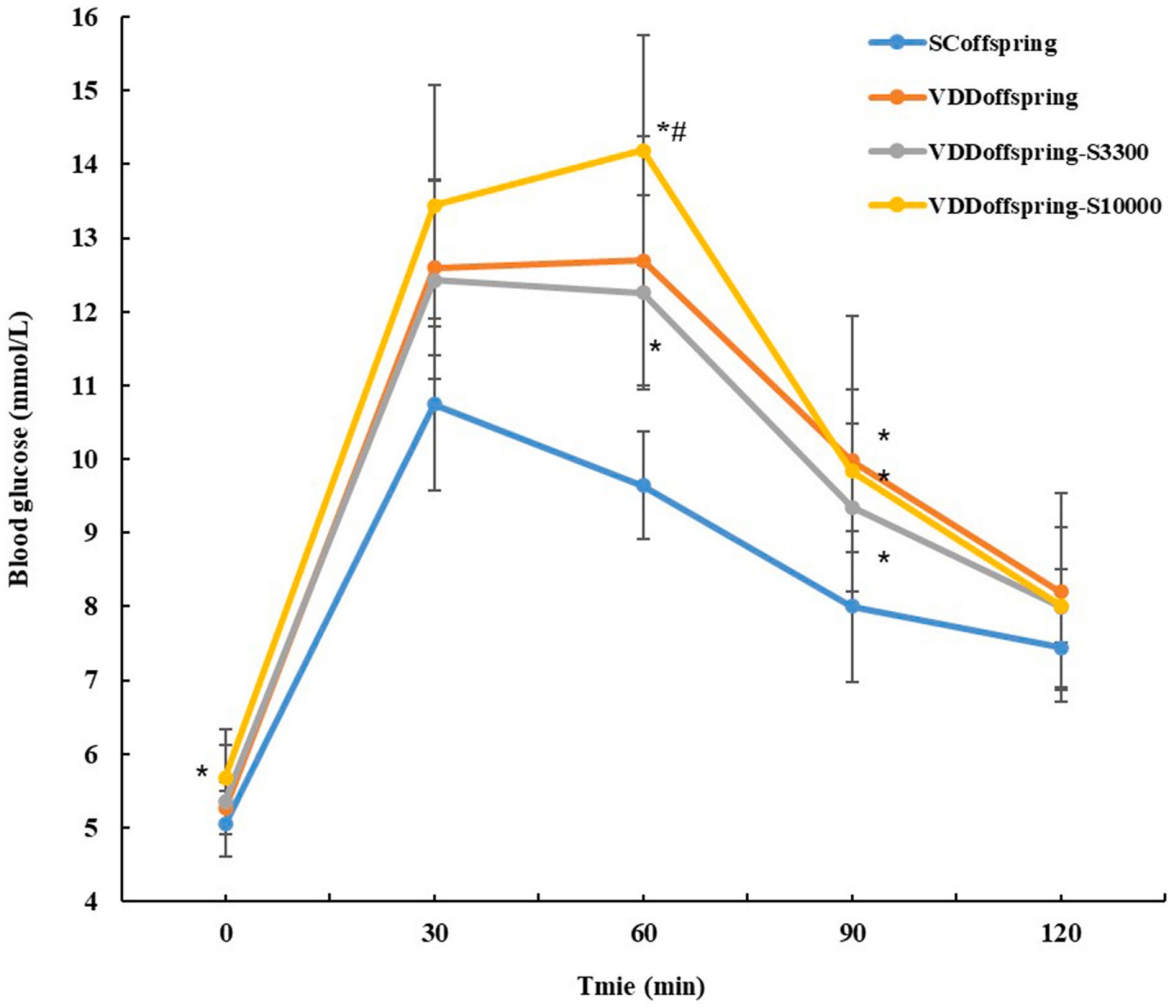
Oral glucose tolerance test (OGTT) in offspring rats aged 70 days (*n* = 8).

Unlike the change in lipid metabolism, vitamin D supplementation at different dosages after weaning had different effects on glucose metabolism and OGTT. Compared with the SC_offspring_ group, the fasting blood glucose levels significantly increased in the VDD_offspring_-S_10000_ group on PND70 (data from OGTT at 0 min) and PND84. Moreover, the levels of FIN and HOMA-IR were obviously increased in the VDD_offspring_-S_33000_ group on PND56, whereas the fasting blood glucose levels in this group remained similar to those in the other groups. As shown in Figure 3, the OGTT showed that the glucose levels of offspring in the VDD_offspring_-S_3300_ group were lowest at all five time points among the VDD_offspring_, VDD_offspring_-S_3300_ and VDD_offspring_-S_10000_ groups, although the levels were still significantly higher than those in the SC_offspring_ group (except at 120 min). In addition, we found that the highest blood glucose levels (fasting glucose level and postprandial blood glucose at 30 min, 60 min, and 90 min) among the four groups were in the VDD_offspring_-S_10000_ group, which indicated that the highest dosages of vitamin D supplementation (10,000 IU) may negatively affect the blood glucose levels in offspring.

## Discussion

Vitamin D deficiency during pregnancy is widespread worldwide, and as vitamin D plays an important role in maintaining normal glycolipid metabolism, the effect of maternal vitamin D deficiency during pregnancy on glycolipid metabolism in offspring has aroused researchers’ attention. Emerging clinical evidence shows that maternal vitamin D deficiency during pregnancy may lead to abnormal growth (either low birth weight or largeness for gestational age) and lipid and glucose metabolism disorders in offspring (23). Some cohort studies showed that lower maternal vitamin D status was associated with higher triglyceride level in infant umbilical arterial blood (12), increased odds of overweight at the first year (24), greater fat mass at ages 4 and 6 years (13, 18). Up to now, however, relevant studies are few, and most of them focused on changes in early childhood, possibly due to follow-up time or confounding factors. Based on the Developmental Origins of Health and Disease (DOHaD) theory, the adverse health effects for their offspring may last longer than expected (23).Thus, taking advantage of animal experiments, we attempt to explore the long-term effects of maternal vitamin D deficiency during pregnancy on glycolipid metabolism of offspring rats.

In this study, we demonstrated an absolutely adverse effect of vitamin D deficiency during pregnancy on the lipid metabolism of their offspring rats in early life. At PND21, higher serum levels of TC, TG and LDL-C and higher ratios of TC/HDL and LDL/HDL were observed in the VDD_offspring_ group than in the SC_offspring_ group. The maternal vitamin D levels during pregnancy were negatively correlated with the levels of TC, TG and LDL-C, as well as with the TC/HDL cholesterol ratio and LDL/HDL cholesterol ratio, in offspring rats. These results were similar to a mother-infant cohort study (12). They determined that compared with the control group (25[OH]D ≥ 20 ng/mL), vitamin D-deficient pregnant women had higher concentrations of TC and lower concentrations of HDL-C in infant umbilical arterial blood, and there was a negative correlation between maternal serum 25(OH)D and the TC level in the umbilical artery. Moreover, linear regression models showed that 81.4% of the TC/HDL cholesterol ratio and 66.8% of the LDL/HDL cholesterol ratio were determined by maternal vitamin D levels during pregnancy for offspring rats at PDN 21. Recent studies have claimed that the TC/HDL and LDL/HDL cholesterol ratios, which can simultaneously evaluate TC, HDL-C and LDL-C levels, were deemed to be better indices for predicting the risk of metabolic-related diseases than a single lipoprotein (25, 26). This suggests that maternal vitamin D deficiency during pregnancy not only causes abnormal lipid metabolism in their offspring in early life but also may increase the risk of metabolic diseases in offspring, including obesity and diabetes. Indeed, in addition to lipid metabolism, the data from the rat weight monitoring and OGTT experiment mentioned below also seemed to support this speculation.

Data from population-based prospective cohort studies showed that maternal vitamin D deficiency significantly increased the risk of neonatal low birth weight and small-for-gestational-age (27–31). Consistent with these studies, we also found significantly lower birth weights in the VDD_offspring_ group than in the SC_offspring_ group. Low birth weight is considered a risk factor for metabolic syndrome in adulthood (32, 33). Interestingly, in addition, we observed that compared with vitamin D sufficiency during pregnancy, prenatal vitamin D deficiency likely accelerated the weight gain of offspring rats before near adulthood and maintained their body weight at a relatively higher level after adulthood. We assumed that the observed accelerated growth at the infantile and juvenile stages may represent “catch-up growth” due to the removal of vitamin D deficiency in the uterus. Data have emerged to suggest that excessive catch-up growth may elicit deleterious effects over a long-term time period (32, 34). Thus, the low birth weight and accelerated preadult weight gain caused by maternal vitamin D deficiency during pregnancy, as well as the possible risk of metabolic disease, should be seriously considered.

Fortunately, rats in the VDD_offspring_ group were given the same dosage of vitamin D (standard dosage: 1,000 IU vitamin D3/kg) as the SC_offspring_ group after weaning, and all of the indices of lipid metabolism, including TC/HDL and LDL/HDL cholesterol ratios and the weight gain of rats, were effectively improved. From PND56, we no longer observed significant differences in body weight between these two groups. After near adulthood, all indices of lipid metabolism and body weight in the VDD_offspring_ group were similar to those in the SC_offspring_ group; however, it cannot be ignored that most of these altered indices were numerically still higher in the VDD_offspring_ group. These results suggested that early supplementation of adequate vitamin D for offspring could ameliorate (rather than completely eliminate) the adverse effects of maternal vitamin D deficiency during pregnancy on lipid metabolism and weight gain.

Unlike lipid metabolism, we did not observe any significant differences in the indices of glucose metabolism (fasting glucose, fasting insulin and HOMA-IR) between the VDD_offspring_ and SC_offspring_ groups at any stage, which is in agreement with the results of other studies (20, 35–38). Some animal experiments to identify the impact of maternal vitamin D deficiency on glucose metabolism in normal and high-fat diets observed glucose homeostasis (fasting glucose, fasting insulin, HOMA-IR and insulin tolerance tests) of adult offspring mice with no modifications in the normal diet; however, the high-fat diet combined with maternal vitamin D deficiency could aggravate the disruption of glucose homeostasis in the male offspring (20, 35). These studies implied that maternal vitamin D deficiency during pregnancy likely had little effect on the baseline glucose metabolism (fasting glucose, fasting insulin and HOMA-IR) of their offspring when they were fed a normal diet; however, it may amplify the adverse effects of other adverse factors on glucose metabolism. In addition, Zhang et al. (37) reported that compared with the control group, vitamin D deficiency during pregnancy in rats led to a marked increase in fasting insulin and HOMA-IR levels at 16 weeks, but no significant differences were observed at 0, 3 and 8 weeks. In addition, glucose metabolism is age dependent. The adverse effects of maternal vitamin D deficiency on the baseline glucose metabolism of the offspring may well be observed after a certain period of accumulation. However, our study was only focused on PND84.

Notably, the OGTT demonstrated significantly higher blood glucose levels in the VDD_offspring_ group at 30 min, 60 min, and 90 min, although blood glucose levels at 0 min and 120 min were similar between the VDD_offspring_ and SC_offspring_ groups. The linear regression model indicated that 35.8, 50.8 and 20.4% of blood glucose levels at 30 min, 60 min, and 90 min were determined by maternal vitamin D levels during pregnancy, respectively. The results indicated that maternal vitamin D deficiency during pregnancy was closely related to postprandial hyperglycemia of the offspring as adults, which was similar to the research of Pei Li et al. (35). In their study, they also found that the number of insulin-positive cells and amount of insulin secretion significantly decreased in the maternal VD-deficient group, which indicated the existence of impaired pancreatic β-cells in the maternal VD-deficient group. Regrettably, we did not measure insulin levels at the time corresponding to OGTT. However, we believe that maternal vitamin D levels during pregnancy likely affect postprandial blood glucose in offspring by facilitating the action and secretion of insulin (39, 40), which requires further studies to confirm.

In view of the important role of vitamin D in metabolic health, more and more vitamin D supplementation intervention studies are conducted. A follow-up study of 988 healthy adolescent girls in Iran showed that significant reductions in serum fasting blood glucose, total- and low-density lipoprotein-cholesterol were observed after high dosages of vitamin D (50,000 IU) supplementation for 9 weeks (41). A meta-analysis including 19 RCTs (1,550 participants) claimed vitamin D supplementation significantly reduced serum fasting plasma glucose, insulin concentration and HOMA-IR in women with GDM (42). Whether supplementation with higher dosages of vitamin D can effectively improve abnormal glycolipid metabolism caused by vitamin D deficiency during pregnancy? Unfortunately, little relevant researches have been done. To clarify this question, we continuously administered medium and high dosages of vitamin D to the offspring of vitamin D-deficient rats during pregnancy (VDD_offspring_-S_3300_ and VDD_offspring_-S_10000_ groups) after weaning.

However, no significant differences were observed in the indices of lipid metabolism and body weight among the VDD_offspring_, VDD_offspring_-S_3300,_ and VDD_offspring_-S_10000_ groups at all of the time points, which indicated that higher dosages of vitamin D supplementation did not further improve the effect of vitamin D deficiency during pregnancy on offspring lipid metabolism and body weight. This result was supported by a meta-analysis of randomized clinical trial. They claimed Vitamin D supplementation (the high dosages were greater than 4,000 IU) cannot effectively decrease the levels of LDL, TC, TG, and BMI (43). Interestingly, in addition, we found that supplementation with different dosages of vitamin D had different effects on improving postprandial hyperglycemia. According to the OGTT, we found that the glucose levels in the VDD_offspring_-S_3300_ group were lowest at all five time points among the VDD_offspring_, VDD_offspring_-S_3300,_ and VDD_offspring_-S_10000_ groups, although they were still significantly higher than those in the SC_offspring_ group. Unexpectedly, the highest dosages of vitamin D supplementation (10,000 IU) negatively affected blood glucose. Rats in the VDD_offspring_-S_10000_ group exhibited the highest blood glucose levels (fasting glucose level and postprandial blood glucose) among the four groups. Although no studies have been conducted on the effects of such high-dosage vitamin D supplementation on glucose metabolism, some other studies have demonstrated a nonlinear association between the oral dosages of vitamin D and disease risk (44–47). Zittermann et al. demonstrated a U-shaped association between major cardiac and cerebrovascular events and a circulating 25(OH)D level of 20–120 nmol/L in cardiac surgical patients (48). A longitudinal analysis from the UK primary care database showed that the hazard ratios for cardiovascular disease and mortality were significantly increased in patients with 25(OH)D < 35 nmol/L or 25(OH)D ≥ 100 nmol/L (44). Therefore, we assumed that there was a nonlinear relationship between vitamin D levels and blood glucose levels on OGTT, and supplementation with 3,300 IU vitamin D may have been the most effective dosage in improving postprandial hyperglycemia in offspring caused by vitamin D deficiency during pregnancy; however, the concentration of vitamin D supplementation still did not reduce postprandial hyperglycemia to the levels in the control group.

According to the results of this study, it is not difficult to find that the adverse effects of vitamin D deficiency during pregnancy on metabolic health of the offspring are long-term, including lipid metabolism abnormalities in early life, low birth weight, faster growth rate before adulthood, higher body weight in adulthood, and higher postprandial blood sugar levels in adulthood. What’s more, giving adequate vitamin D supplementation to offspring after weaning can improve some adverse health outcomes, but it cannot completely eliminate them. This means timely and sufficient vitamin D intervention during pregnancy may be more effective than nutritional intervention in childhood. Thus, it is necessary for relevant departments to develop effective measures to increase the proportion of vitamin D sufficiency in women of childbearing age. In addition, there were some limitations in this study. First, the gender of the offspring was not classified, and the effects of maternal vitamin D deficiency during pregnancy may be different in male and female offspring. Second, considering that glucose metabolism is age-dependent, the effects of vitamin D deficiency during pregnancy on glucose metabolism in offspring may require longer observation times. We speculate that these factors should be considered in future studies.

## Conclusion

In summary, hypovitaminosis D during pregnancy had different adverse effects on the body weight and glycolipid metabolism of the offspring at different stages compared to maternal vitamin D sufficiency. The adverse effects could indicate a higher risk of metabolic disease for offspring in adulthood, such as obesity and diabetes. Over a long period of time, adequate vitamin D supplementation after weaning is beneficial to glycolipid metabolism in the offspring of maternal vitamin D deficiency during pregnancy; however, the improvement is incomplete. In addition, higher dosages of vitamin D supplementation after weaning do little to improve these adverse effects (except for postprandial hyperglycemia). Therefore, maintaining optimal vitamin D status during pregnancy is especially important for the health of the offspring.

## Data availability statement

The original contributions presented in the study are included in the article/Supplementary material, further inquiries can be directed to the corresponding author.

## Ethics statement

All experimental procedures followed the guidelines for the care and use of animals, which were established by Zhejiang Chinese Medical University and approved by the Animal Experimentation Ethics Committee of Zhejiang Chinese Medical University (Approval NO. IACUC-20200518-15).

## Author contributions

ZC, TW, XQ, and YH were responsible for experimental operation. ZC was responsible for writing the first draft of the article and analyzed the data with YZ. WH revised and finalized the article. All authors contributed to the article and approved the submitted version.

## Funding

This work received financial support from the Basic Public Welfare Research Project Zhejiang Province (grant number: GD20H260003), Social Development Scientific Research Projects of the Science and Technology Bureau of Hangzhou (2020ZDSJ0402) and Science and Technology Program of Medicine and Health of Hangzhou (0020190275). The funding institution had no role in the design, data collection, analysis, interpretation, and writing of the manuscript.

## Conflict of interest

The authors declare that the research was conducted in the absence of any commercial or financial relationships that could be construed as a potential conflict of interest.

## Publisher’s note

All claims expressed in this article are solely those of the authors and do not necessarily represent those of their affiliated organizations, or those of the publisher, the editors and the reviewers. Any product that may be evaluated in this article, or claim that may be made by its manufacturer, is not guaranteed or endorsed by the publisher.
