# Supplementary material for: The effect of maternal vitamin D deficiency during pregnancy on glycolipid metabolism of offspring rats and the improvement of vitamin D intervention after weaning
Source: Front Nutr. 2023 Jul 31;10:1214040. doi: 10.3389/fnut.2023.1214040 (PMC10426798; doi:10.3389/fnut.2023.1214040)
Supplement: Supplementary file 1 [file Data_Sheet_1.docx]

Supplementary Table 1 The serum 25(OH)D concentration and calcium levels of offspring rats among different groups at different time points

| Group | PND21 | |  | PND56 | |  | PND84 | |
| --- | --- | --- | --- | --- | --- | --- | --- | --- |
|  | 25(OH)D (μg/ml) | calcium (mmol/L) |  | 25(OH)D (μg/ml) | calcium (mmol/L) |  | 25(OH)D (μg/ml) | calcium (mmol/L) |
| SCoffspring （n=8) | 7.35±0.85 | 2.32±0.06 |  | 11.48±1.82 | 2.35±0.05 |  | 11.11±1.82 | 2.34±0.06 |
| VDDoffspring （n=8) | 7.73±2.19 | 2.28±0.04 |  | 11.55±3.58 | 2.34±0.07 |  | 10.72±1.52 | 2.31±0.04 |
| VDDoffspring-S3300 (n=8) | - | - |  | 21.98±5.88 | 2.39±0.05 |  | 21.85±3.19 | 2.30±0.06 |
| VDDoffspring-S10000 (n=8) | - | - |  | 45.66±7.55 | 2.34±0.07 |  | 50.34±15.73 | 2.35±0.05 |
| P value | 0.650 | 0.174 |  | <0.001 | 0.290 |  | <0.001 | 0.234 |

Supplementary Table 2 The effect of maternal vitamin D levels during pregnancy (E18) on lipid metabolism (PND21) and OGTT of offspring rats

| Dependent variable | coefficient of determination (R^2^) | p value | Regression equation |
| --- | --- | --- | --- |
| TC | 0.343 | 0.017 | Y=2.565-0.041X |
| TG | 0.254 | 0.046 | Y=0.994-0.025X |
| LDL | 0.433 | 0.006 | Y=0.589-0.014X |
| TC/HDL | 0.814 | <0.001 | Y=4.442-0.096X |
| LDL/HDL | 0.668 | <0.001 | Y=1.017-0.029X |
| Blood glucose (30min) | 0.358 | 0.019 | Y=13.38-0.26X |
| Blood glucose (60min) | 0.508 | 0.005 | Y=13.84-0.407X |
| Blood glucose (90min) | 0.204 | 0.013 | Y=10.658-0.253X |
